# Detection of neuronal defensive discharge information transmission and characteristics in periaqueductal gray double-subregions using PtNP/PEDOT:PSS modified microelectrode arrays

**DOI:** 10.1038/s41378-023-00546-8

**Published:** 2023-05-31

**Authors:** Botao Lu, Penghui Fan, Ming Li, Yiding Wang, Wei Liang, Gucheng Yang, Fan Mo, Zhaojie Xu, Jin Shan, Yilin Song, Juntao Liu, Yirong Wu, Xinxia Cai

**Affiliations:** 1 State Key Laboratory of Transducer Technology, Aerospace Information Research Institute, Chinese Academy of Sciences, Beijing, 100190 China; 2 University of Chinese Academy of Sciences, Beijing, 100049 China

**Keywords:** Biosensors, Nanofabrication and nanopatterning

## Abstract

Threatened animals respond with appropriate defensive behaviors to survive. It has been accepted that midbrain periaqueductal gray (PAG) plays an essential role in the circuitry system and organizes defensive behavioral responses. However, the role and correlation of different PAG subregions in the expression of different defensive behaviors remain largely unexplored. Here, we designed and manufactured a microelectrode array (MEA) to simultaneously detect the activities of dPAG and vPAG neurons in freely behaving rats. To improve the detection performance of the MEAs, PtNP/PEDOT:PSS nanocomposites were modified onto the MEAs. Subsequently, the predator odor was used to induce the rat’s innate fear, and the changes and information transmission in neuronal activities were detected in the dPAG and vPAG. Our results showed that the dPAG and vPAG participated in innate fear, but the activation degree was distinct in different defense behaviors. During flight, neuronal responses were stronger and earlier in the dPAG than the vPAG, while vPAG neurons responded more strongly during freezing. By applying high-performance MEA, it was revealed that neural information spread from the activated dPAG to the weakly activated vPAG. Our research also revealed that dPAG and vPAG neurons exhibited different defensive discharge characteristics, and dPAG neurons participated in the regulation of defense responses with burst-firing patterns. The slow activation and continuous firing of vPAG neurons cooresponded with the regulation of long-term freezing responses. The results demonstrated the important role of PAG neuronal activities in controlling different aspects of defensive behaviors and provided novel insights for investigating defense from the electrophysiological perspective.

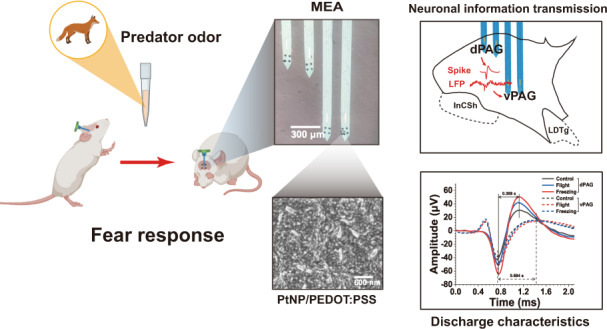

## Introduction

Fear is a conservative state in the process of biological evolution that triggers defensive behavior to effectively avoid or reduce potential damage to animals^1,2^. After encountering predators, rodents express different defensive behavior patterns according to surrounding factors and risk of threat, such as the presence of shelters^3^. When predators and attack are highly imminent in the environment, rodents present a circa-strike defense (jumping and flight). As emerging threats decrease, rodents change to a freezing state^4,5^. Many brain regions are involved in different aspects and levels of defensive behavior control. These regions include but are not limited to the cortex, hypothalamus, central amygdala (CEA), and continuous strip of midbrain structures, which collectively generate innate fear and execute specific defensive actions^6–9^.

Previous studies have demonstrated that the periaqueductal gray (PAG) is the critical node of coordinated survival in the central network, which mediates fear-evoked and defensive behaviors^10,11^. Anatomically, the PAG is organized in longitudinally organized functional columns, consisting of the ventral PAG (vPAG), which are separated from the dorsal PAG (dPAG) by the lateral column^12,13^. There is sufficient evidence that the dPAG participates in the motor and autonomous response of defensive states as follows: electrical stimulation of the dPAG can induce aversion reactions, such as freezing and escape, in rats^14,15^. Conversely, the vPAG seems to be the indispensable structure to express conditioned fear and learned behavior, and lesions of the vPAG reduced the freezing reaction to the neutral stimulus with footshock but did not affect the flight response^16–18^. PAG receives inputs from crucial brain regions involved in fear regulation, such as the hypothalamus^19^ and CEA^20^. However, the functional role of the PAG subregion in the expression of active and passive defensive behaviors remains poorly understood, including the firing characteristics, exact functional role of PAG neurons in different subregions, neuronal microcircuitry and correlation in the PAG subregion during the expression of different predator odor-induced defensive behaviors^8^.

In the existing research on the relationship between animal behavior and neural activities in different regions of the brain, traditional nerve detection electrodes that lacked spatial and temporal resolution, including metal wire and single channel electrodes^21^, could not identify and functionally characterize the interactions between multiple regions^22^. With the development of microelectromechanical system (MEMS) technology, implantable microelectrode arrays are widely used in neural information recording due to their long-term stability and high resolution^23^. To reduce brain tissue damage and improve the detection performance of MEA^24^, the surface of MEA is usually modified with composite conductive materials as functional layers. Poly(3,4-ethylene dioxythiophene): poly(styrene sulfonate) (PEDOT:PSS) is a conductive polyelectrolyte complex with good biological compatibility and electrical stability^25–27^. Although PEDOT:PSS exhibits many excellent characteristics when applied to the neural interface, some studies have found that its adhesion to metal electrodes and mechanical stability are poor, which is not conducive to the long-term detection of the neural interface. Platinum nanoparticles (PtNPs) can provide rough and stable surface structures for electrode surfaces^28^. In this study, PtNP and PEDOT:PSS composite coatings were used to improve the adhesion area and adhesion of PEDOT:PSS, which was helpful for improving the quality of recorded signals and improved the signal-to-noise ratio (SNR) and long-term detection performance.

The development of MEAs with multiple channels and multiple brain regions promotes research on fear-related neural circuit mechanisms and fear-related diseases^29,30^. Based on the above, we designed a kind of MEA with high spatial and temporal resolution to simultaneously record neuronal information in the dPAG and vPAG of rats with innate fear and modified the surface of microelectrodes with a PtNP/PEDOT:PSS nanocomposite. Combined with the changes in rat defense behavior output and the neural dynamics of the dPAG and vPAG recorded by MEA, we explored the changes in neuronal correlation and activity mode of the dPAG and vPAG in the process of defense behavior. The results showed that both the dPAG and vPAG were involved in the regulation of defensive behavior induced by innate fear in rats. We found that the firing patterns of dPAG and vPAG neurons were significantly different, and dPAG neurons participated in active defense regulation with explosive firing patterns. Overall, our research elucidated the integration mechanism for the participation of the dPAG and vPAG in defense behavior from the perspective of electrophysiology. In addition, we discussed the influence of neuronal firing patterns on the dPAG and VPAG participating in different defense behaviors, and provided new ideas for studying PAG subregion relationships and fear-related diseases.

## Materials and methods

### Reagents and apparatus

2-Methylthiazoline (2MT) was obtained from Shanghai Acmec Biochemical Co., Ltd. (China). Chloroplatinic acid (H_2_PtCl_6_) and lead acetate [Pb(CH_3_COO)_2_] were obtained from Sinopharm Chemical Reagent Co., Ltd. (China). 3,4-ethylene dioxythiophene (EDOT) was purchased from Shanghai Aladdin Biochemical Technology Co., Ltd. (China). PSS was obtained from Shanghai Herochem Co. Ltd. (China).

The stereotactic apparatus (51600) was purchased from Stoelting (USA). A 128-channel neuroelectrophysiological recording system was purchased from Blackrock Microsystems (USA). Micropositioner (Model 2662) was purchased from David KOPF instrument (USA).

### Fabrication and nanomodification of the MEA

Based on the demand for dual brain region detection, we prepared a 16-channel MEA array for the detection of dPAG and vPAG in conscious rats in the fear state. To synchronously detect neuronal information transmission and changes in the dPAG and vPAG in real time, we designed an MEA based on the anatomical structure and relative position of the dPAG and vPAG (Fig. S2a). The distribution of recording sites covered as many vPAG and dPAG regions as possible, which was conducive to detecting broader neuronal signals and precisely implanting MEA to reduce brain tissue injury in the target region. The lengths of the four shanks were 8.5, 8.5, 7.7, and 7.6 mm. The distance between the shanks was 110 mm. Each microelectrode can be used for the detection of electrophysiological signals. The manufacturing process adopted MEMS technology. A brief description is provided below with the MEA fabrication in Fig. S1.

The MEA was fabricated with a silicon-on-insulator (SOI) wafer (25 µm Si/1 µm SiO_2_/550 µm Si) substrate, and a 200 nm layer of SiO_2_ was deposited on the substrate to insulate devices from the SOI substrate. A layer of photoresist was spin-coated on the wafer. This was followed by sputtering 250 nm Pt and 30 nm Ti as the metal layer and lift-off processing to form recording sites, pads, and microwires. Next, an insulating layer (500 nm Si_3_N_4_/300 nm SiO_2_) was deposited. Then, the MEA was shaped by photolithography and reactive ion etching (RIE). Finally, individual MEAs were separated by KOH (80 °C, 30%) back wet etching of the SOI wafer.

After the MEAs were fabricated, we modified the surface of all recording sites with PtNPs/PEDOT:PSS to improve the detection performance and biocompatibility of the MEAs. The PtNP layer was deposited on recording sites by the chronoamperometric method (CA, −1.0 V, 60 s) in the electroplating solution involving 48 mM chloroplatinic acid and 4.2 mM lead acetate. Then, 0.1 M PSS solution and 20 mM EDOT were mixed for 1 h ultrasonic dispersion. The deposition was carried out by cyclic voltammetry (CV) between 0 and 0.95 V at a rate of 50 mV/s for 10 cycles in the mixed solution. The electroplating of PtNPs/PEDOT:PSS on the surface of microelectrodes was realized.

### Animals and surgical procedures

All animal experiments were conducted with the permission of the Beijing Association on Laboratory Animal Care and approved by the Institutional Animal Care and Use Committee at the Aerospace Information Research Institute, Chinese Academy of Science (AIRCAS). A total of 15 adult male Sprague‒Dawley (SD) rats (230 ~ 350 g, 8–12-week-old; Vital River Laboratory Animal Technology Co., Ltd, Beijing, China) were used in this study. All rats were housed in a temperature-controlled environment (22 ~ 26 °C and 50 ~ 80% humidity) maintained on a 12/12 h dark/light cycle. Food and water were available *ad libitum*.

**Fig. 1:**
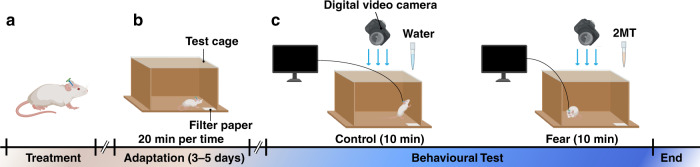
**Schematic diagram of behavior and electrophysiological activity records of 2MT-induced defense behavior. a** The MEA was fixed on the rat head with dental cement. **b** Each rat underwent adaptive training for 20 min each time for 3–5 days in the test cage. **c** Schematic diagram of innate fear induced by 2-MT in rats. (*n* = 15)

The rats were deeply anesthetized with 5% isoflurane in oxygen-enriched air before the surgical operation. Each rat was fixed in a stereotactic apparatus with nontraumatic ear bars and was anesthetized with 1–2% isoflurane in oxygen-enriched conditions. During the operation, the depth of anesthesia was checked regularly by examining the corneal and paw withdrawal reflexes. An incision was cut from the midline scalp to expose the skull. After drilling a 2 × 2 mm hole in the skull, MEA was implanted into PAG (AP: –7 mm, ML: –0.9 mm) at a constant speed of 2 μm/s using a micropositioner and paused for 2~3 min every 200 μm implanted. The MEA was attached to the skull with 5 screws and dental cement (Fig. 1a). To improve the detection SNR, metal wires were used to connect the ground with skulls and MEA. After craniotomy, the rats were intraperitoneally injected with 45,000 units of penicillin to reduce inflammation. The rats recovered for 3~5 days before the start of behavioral testing (Fig. 1b). During the adaptation period, we placed the rats gently in the center of the test box every day and the rats could explore freely in the experimental box for 20 min to fully adapt to the experimental environment. The room remained quiet and consistent with the experimental conditions during the adaptation process.

### A assay of 2MT-evoked innate fear

Odor extracts or analogs from the fur and feces of predators have been widely used to stimulate the innate fear of rats^31^. Fox odor 2,4,5-trimethyl thiazoline (TMT) and 2-Methylthiazoline (2MT) are the most widely used efficient predator odors, which trigger strong defensive behavior in rats^32–34^. In this study, 2-MT was used to induce innate fear in rats (Fig. 1c). During the experiment, we used a digital video camera (30 frames/s, 1080 × 1920 pixels) and a 128-channel neuroelectrophysiological recording system to simultaneously record the behavior and neuroelectrophysiological activities of rats. Predator odor was introduced into the test cage by gently dropping water or 2-MT onto the filter paper placed in the corner of the test cage. To reduce external electromagnetic interference, the outside of the test cage was covered with a metal screen and grounded. Before the experiment, rats had 5 ~ 10 min of free time to adapt to the test cage. First, 80 μL of water was gently added onto the filter paper. The rats could freely explore the test cage for 10 min. After 10 min, 80 μL of 2MT was dripped onto the filter paper for 10 min. The behavior of rats was monitored by the digital camera above the test cage. After the experiment, we used 75% alcohol and water to deeply clean the test chamber and dried it to prevent the smell of the previous experiment from affecting the next experiment.

Ethvision (Blackrock Microsystems, Salt Lake, UT, USA) behavior analysis software was used to postprocess the captured video. If the rat had not moved for move than 2 s, the rat entered the freezing state. The freezing rate was the percentage of freezing time in the total duration, which was used to measure the freezing degree and was independently analyzed by computer software. The software could automatically obtain the rats’ body center to calculate the position and movement speed. The neural signals were collected by preamplifier and differential amplifier circuits, which were divided into action potential spikes and local field potentials (LFPs). The digital sampling rate of the spike signal was 30 kHz, and it was extracted by a 200 Hz high-pass filter. Spike waveform characteristics, including amplitude, peak interval, and power spectrum, were derived from the sampled waveform by clustering K-mean analysis. The sampling rate of the raw LFP data was 1 kHz. The LFP was extracted from the raw LFP data by using a 200 Hz low-pass filter, with the ground as the reference (Fig. S3).

### Histology

After the behavioral tests, the animals were deeply anesthetized and terminated by transcardial perfusion (0.9% sodium chloride and 4% paraformaldehyde). The brains were extracted and postfixed with 20% sucrose solution overnight at 4 °C. After postfixation, the brain was cryoprotected in 30% sucrose solution. Coronal sections were cut into 50 μm sections with a cryotome. Figure S2b and c shows the histological validation diagram of the target location. The coronal section demonstrated that the short handle was implanted in the dPAG, and the long handle was implanted in the vPAG. The neuronal information activities of dPAG and vPAG neurons could be detected synchronously by MEA after implantation.

### Statistical methods

All neuroelectrophysiological data were analyzed offline with NeuroExplorer software (NexTechnologies, USA) and OfflineSorter (Plexon, USA). ORIGIN (OriginLab, USA) and MATLAB (The Mathworks, USA) were used for statistical analysis. Unless otherwise specially illustrated, values are expressed as the mean ± SEM. Significance tests of different groups were determined using Student’s t-test or one-way ANOVA with Tukey’s *post hoc tests*. Significance levels are indicated as follows: *p < 0.05; **p < 0.01; ***p < 0.001; ns was not significant (*p* > 0.05).

The trough-to-right peak latency was calculated from the time interval between the peak and trough of the average peak waveform. The spike asymmetry index was the ratio of the difference between the right and left trough-to-peak amplitudes of the average peak waveform and their sum^35,36^.

## Result

### Electrical performance characterization of MEAs

**Fig. 2:**
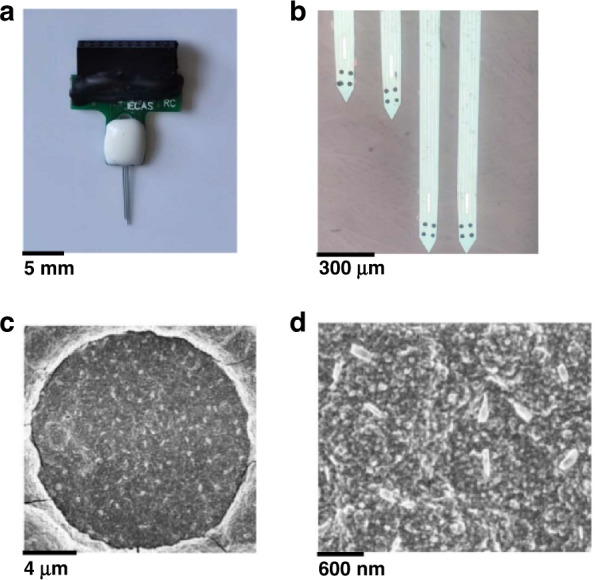
**PtNP/PEDOT:PSS-modified MEAs. a** MEA packaged with the printed circuit board. **b** Microscopic image of the MEA tip modified with PtNP/PEDOT:PSS. **c** PtNP/PEDOT:PSS-modified microelectrode surface under scanning electron microscopy (SEM). **d** SEM image of the PtNPs/PEDOT:PSS nanocomposites

Figure 2a, b show the packaged MEA and an enlarged view of the modified MEA tip under the microscope. Black modification materials were uniformly distributed on each microelectrode site. The scanning electron microscopy (SEM) image indicated that the PtNP/PEDOT:PSS nanocomposite adhered closely to the surface of the microelectrode (Fig. 2c, d). The PtNPs improved the roughness of the microelectrode, and the PEDOT:PSS structure covered the entire surface of the electrode, which was conducive to improving the stability of the electrode. The modification of PEDOT also improved the biocompatibility and long-term detection capability of microelectrodes in vivo. High-quality neuroelectrophysiological signals could still be detected after being implanted in rats for >40 days (Fig. S4).

**Fig. 3:**
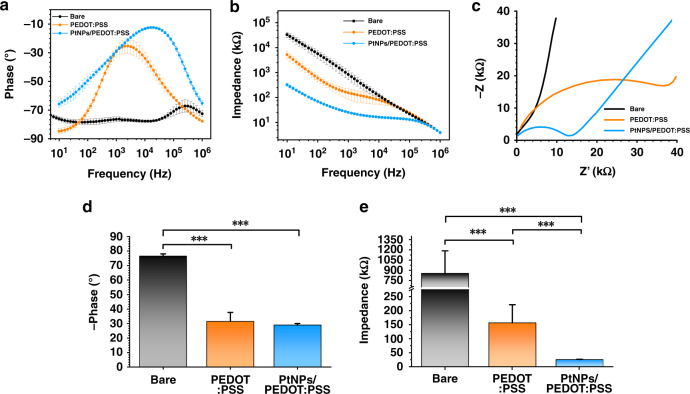
**Electrical performance characterization of MEA. a** Impedance characteristics of bare electrodes, PEDOT:PSS-modified microelectrodes, and PtNP/PEDOT:PSS-modified microelectrodes at 10–1,000,000 Hz. **b** Phase delay of bare electrodes and microelectrodes with different modified materials at a frequency of 10–1,000,000 Hz. **c** Nyquist plots of the complex plane impedance spectra of the microelectrode modified by the bare electrode and different modified materials. **d** Impedance and **e** phase delay of the bare microelectrode, PEDOT:PSS-modified microelectrode, and PtNP/PEDOT:PSS-modified microelectrode at 1 kHz frequency. (*n* = 16)

We compared the electrical performance of bare microelectrodes and modified microelectrodes in frequencies from 10 Hz to 1 MHz. The phase delay and impedance of the bare microelectrode were both large in the frequency domain from 10 Hz to 1 MHz (Fig. 3a, b), which was not conducive to the detection of electrophysiological signals in vivo. The phase delay and impedance of the PtNP/PEDOT:PSS-modified microelectrodes were significantly better than those of the bare microelectrodes and the PEDOT:PSS-modified microelectrode at 10 Hz to 1 MHz. The central frequency of the neural activities is ~1 kHz, at which the impedance and phase delay of the three were compared in this work. The microelectrode impedance decreased from 847.07 ± 334.3 kΩ (bare) to 156.18 ± 64.14 kΩ (PEDOT:PSS) and then to 25.55 ± 10.33 kΩ (PtNPs/PEDOT:PSS) (Fig. 3d). There was no significant difference in phase between PEDOT:PSS-modified microelectrodes (-31.41 ± 6.28°) and PtNP/PEDOT:PSS-modified microelectrodes (-28.89 ± 1.15°), which indicated that PEDOT played a major role in phase delay (Fig. 3e). In addition, the Nyquist plots of the impedance spectra showed that the PtNP/PEDOT:PSS-modified microelectrode exhibited intense diffusion characteristics (Fig. 3c). Our results indicated that the PtNP/PEDOT:PSS-modified microelectrodes achieved better electrical performance and biocompatibility, which was helpful for the detection of neuronal information transmission and discharge characteristics.

### Characterization of behavioral and electrophysiological signals during 2MT-induced defensive behavior

**Fig. 4:**
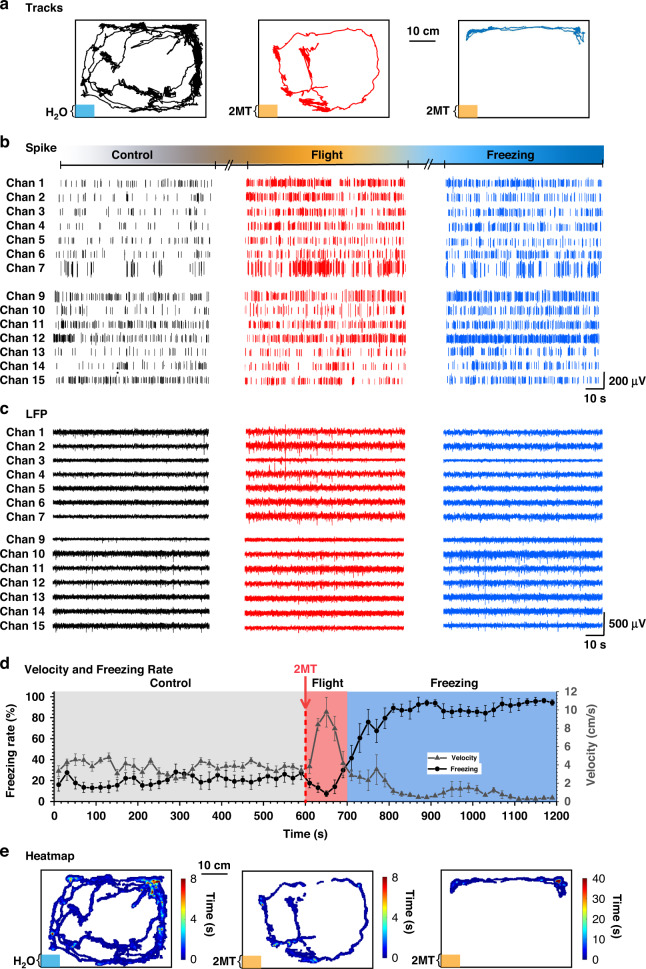
**Recording of behavioral and electrophysiological activities during 2MT-induced defense behavior in rats. a** Real-time movement trace of a representative 2MT-exposed rat in the test cage. The lower left area indicates the location of the filter paper containing water (blue) and 2MT (orange). **b** Real-time recordings of spikes and **c** LFPs of 7 typical channel dPAG (Chan 1–7) and vPAG (Chan 9–15) neurons. **d** The moving velocity and freezing rate of rats during the experiment. Each data point was the velocity and freezing rate within 10 s (*n* = 15). **e** Heatmap of a representative 2MT-exposed rat in the test cage

To examine the role of the dPAG and vPAG in defense behaviors related to innate fear, we combined the electrophysiological activities of dPAG and vPAG neurons recorded by MEA with the behavior changes recorded by the digital camera before and during the defense behavior evoked by exposure to 2MT. We associated the changes in motor output related to defense behaviors with their neural activity patterns, which allowed us to evaluate the endogenous neural mechanism of the dPAG and vPAG under different defense behaviors. After exposure to 2MT, we observed two different innate defense behaviors in rats (Fig. 4a, d). Velocity and freezing rate are important indices of defense behavior (flight and freezing) in rats. The rats showed an obvious flight avoidance reaction, which was characterized by an extremely low freezing rate and high moving velocity. We define this state as flight. Then, after the rats found that escape was impossible, they stopped running, remained almost motionless and began to enter the freezing state. Due to this freezing mechanism, the prey were more likely to avoid capture when threatened. As shown in Fig. 4e, we also generated a temporal heatmap of the spatial position of the rat. The rats in the control group moved evenly throughout the space, and the rats in the flight group circled the space. When rats entered the freezing stage, they stayed far away from the filter paper (the source of predator odor) for a long time.

Our fabricated MEA was a brain-computer interface (BCI) with a high spatiotemporal resolution that provided a powerful strategy for monitoring real-time neuronal electrophysiological activities. The recording sites of MEA closely contacted the neurons in the target area, so the neural information recorded in each channel could accurately reflect the neuronal activities. Fig. 4b, c, and S5 show the typical neural spikes and LFPs recorded in the same rat during the experiment. Compared with the control, the firing density of spikes was significantly more intensive and LFP exhibited higher amplitude and frequent fluctuation during flight in the dPAG; in addition, the spike firing and LFP amplitude during freezing were between the control and flight. The difference was that in the vPAG, the release density of spikes was the most intensive in freezing, and the fluctuation and amplitude of LFPs increased to the most frequent. The above results indicate that the dPAG and vPAG have different activation levels in flight and freezing.

In conclusion, the above results suggested that the highly efficient predator odor analog 2MT induced innate fear (defense) behaviors in rats. Both the dPAG and vPAG were significantly activated during defensive behavior. The dPAG was highly activated during flight, while the vPAG was highly activated during freezing.

### Information transmission and functional analysis of vpag and dpag neurons in the defense state

**Fig. 5:**
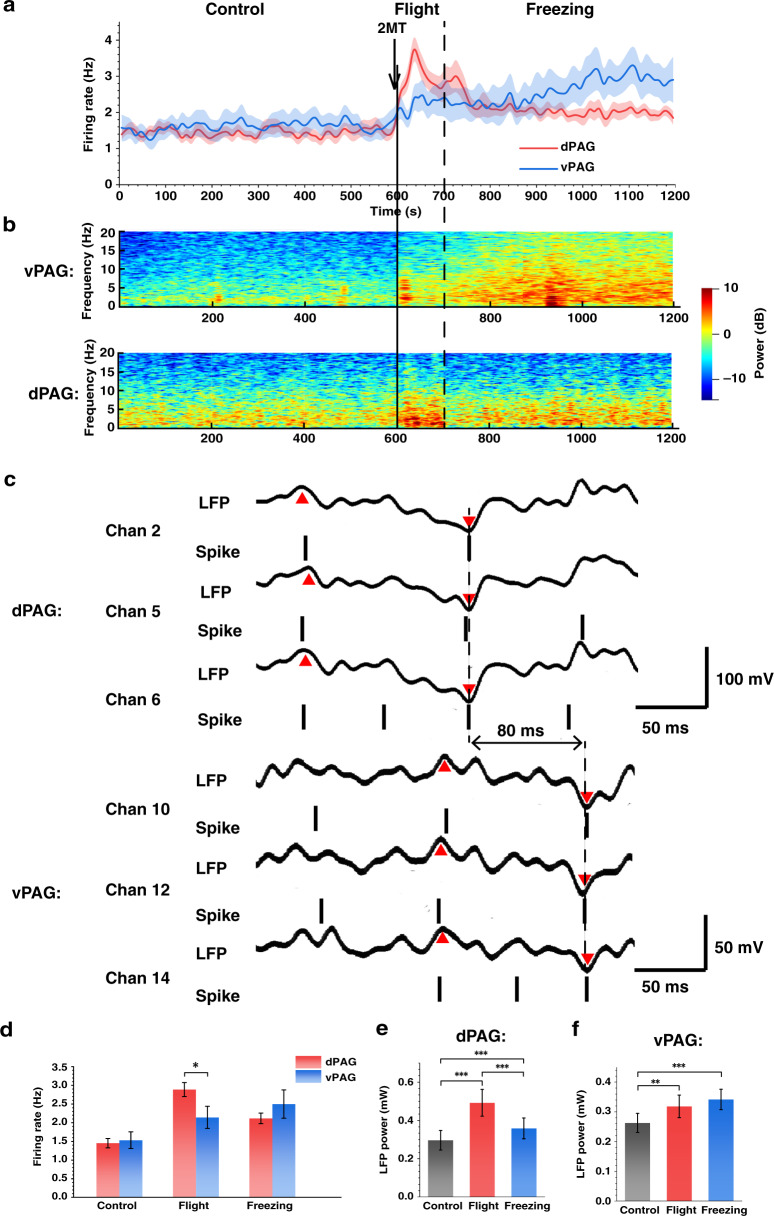
**Analysis of LFPs and spikes in 2MT-induced rat defense behavior. a** The average spike firing rate of dPAG and vPAG neurons during the experiment. Each data point was the average spike firing rate of dPAG or vPAG neurons within 20 s. **b** Representative spectrogram of LFPs in typical channels of the dPAG and vPAG. **C** The relative time of the peak and trough of the LFP waveform and spike firing showed that the discharge spreads from the dPAG to the vPAG. Spike firing only indicated the firing time, not the amplitude. **d** Average spike firing rate and **c** freezing rate of rats in different states. **e** The average LFP power of the dPAG and **f** vPAG under different states

To explore the activities of dPAG and vPAG neurons in the whole process of defensive behavior, we counted the change in average spike firing rate during the experiment (Fig. 5a). After exposure to 2MT, the spike firing rate of dPAG neurons peaked during flight and gradually decreased and stabilized after the peak. This result indicated that the dPAG was rapidly activated to a high degree after exposure to 2MT, and then the activation degree was gradually stable during freezing. The spike firing rate of vPAG neurons began to increase after exposure to 2MT, which indicated that the activation degree of vPAG neurons gradually increased after exposure to 2MT. LFP reflects the real-time transmission of information across the neural network and is an important supplement to measure neural information activity. The energy change in the dPAG and vPAG LFP spectrograms is consistent with the changing trend of the spike firing rate in the defense state (Fig. 5b). To analyze the correlation and spread of defense behavior regulation in PAG subregions, the time when peaks of LFP and spike firing occurred in the dPAG and vPAG were compared after 2MT-induced innate fear (Fig. 5c). LFP peak and spike firing first appeared in the dPAG and were gradually transmitted to the vPAG. In addition, the LFP waveforms of the dPAG and vPAG showed roughly the same components. Transmission was found in multiple channels and accompanied by the release of spikes. The transmission time between typical channels was ~80 ms.

As shown in Fig. 5d, the difference in the average spike firing rate of the dPAG and vPAG was not significant during the control test. During flight, the average spike firing rate of dPAG and vPAG neurons increased significantly, but compared to vPAG neurons, dPAG neurons exhibited significantly higher rates. During freezing, the average firing rate of vPAG neurons continued to increase, while that of dPAG neurons decreased compared with flight and was lower than that of vPAG neurons. The average power of LFPs in the dPAG and vPAG was calculated during different behaviors. Compared with the control state, the average LFP power of the dPAG in the flight and freezing states increased significantly, the LFP power in the flight state increased to 0.49 ± 0.07 mW, and the LFP power in the freezing state decreased to 0.36 ± 0.05 (Fig. 5e). In the vPAG (Fig. 5f), the average LFP power in the flight and freezing states increased significantly compared with the control, and the LFP power in the freezing state (0.34 ± 0.03 mW) was slightly higher, but there was no significant difference compared with the flight state (0.32 ± 0.04 mW). Then, we normalized the power of LFPs in three states and analyzed their relative characteristics in the 0–20 Hz range. We divided it into the delta frequency band (1–4 Hz), theta frequency band (4–8 Hz), alpha frequency band (8–13 Hz), and beta frequency band (13–30 Hz) (Fig. S6b). After exposure to 2MT, the energy of the dPAG was more concentrated in the delta band, and the proportion of the delta band in the flight group was significantly higher than that in the freezing and control groups, while the change in the vPAG was reflected in the increase in the proportion of the theta band.

The results showed that the rapid activation of the dPAG was consistent with flight behavior after exposure to 2MT and accompanied by the transmission of neuronal defense information from the dPAG to the vPAG. When the rats entered the freezing state, the activation degree of vPAG neurons gradually increased, and the activation degree of dPAG neurons began to decline. The spike firing rate of vPAG neurons was highly correlated with the freezing rate (Fig. S7). In other words, the activities of the dPAG may be more related to sports and the active defense response, while the vPAG was consistent with passive defense.

### Comparison and analysis of the neuron spike characteristics of dPAG and vPAG

**Fig. 6:**
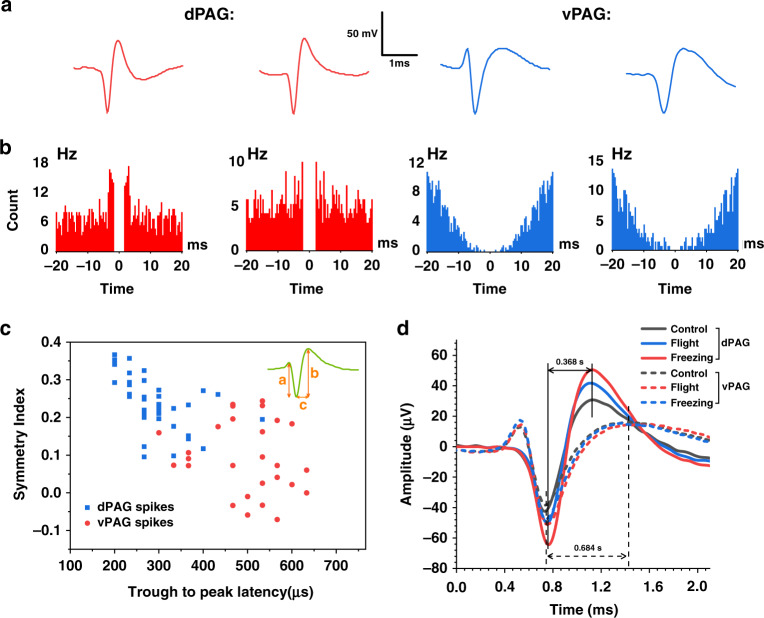
**Differential firing patterns of dPAG and vPAG neurons. a** Average spike waveform (*n* = 84) and **b** spike-time autocorrelograms of the respective units. **c** The neurons are clustered according to waveform asymmetry and the average filtered peak width. Different color symbols represent neuron units in different brain regions. Symmetry index = (*b* − *a*)/(*a* + *b*). **d** Average spike waveform of two typical channel neurons in different states

As we detected that dPAG and vPAG neurons show different activation degrees and exhibited different regulation modes during flight and freezing, this work compared and analyzed the diverse firing patterns of the two types of neurons in defense information transmission. The signal of 84 channels recorded was separated into spike units using principal component analysis (PCA) and valley searching methods (VSM). We calculated the average waveform of individual spike units and analyzed their autocorrelation. As shown in Fig. 6a, the average waveform of dPAG neurons had shorter peak intervals and a narrower waveform than that of vPAG neurons. The spike-time autocorrelogram of dPAG neurons had a shorter latency to firing than that of vPAG neurons (Fig. 6b). Pooling all dPAG and vPAG neurons, we calculated the trough to right peak latency and peak amplitude asymmetry of the average waveform (see “Statistical methods” for the calculation method). Fig. 6c shows that there was a clear distinction between the waveform types of dPAG and vPAG neurons. The trough-to-right peak latency of the dPAG neurons’ average waveform was generally shorter than that of vPAG neurons, and the spike asymmetry index was higher.

In summary, the firing characteristics of dPAG and vPAG neurons were obviously different. The waveform of dPAG neurons was narrow, and the spike-time autocorrelogram latency period was shorter, which also confirms that dPAG neurons were more likely to exhibit burst or burst-like firings; thus, physiological conditions were provided for controlling flight behavior that requires an instant response.

### Changes in spike characteristics of neurons in different defense states

The spike-firing characteristics of neurons provide the physiological basis for neurons in the regulation of defense behavior. To study the characteristic changes in neuronal spikes under different defense states, we also calculated the average spike waveform under different behavior states (Fig. 6d). After exposure to 2MT, the average amplitude of dPAG neuron spikes during control was significantly lower than during flight and freezing. The amplitude was the highest during flight, and the amplitude during freezing was between flight and control. For the vPAG average spike waveform, the amplitudes of the flight and freezing states were similar and were slightly greater than that of the control state. The above changes confirmed that the waveform of PAG neurons changed significantly under the defensive state. Notably, dPAG neurons showed more significant waveform changes, which seemed to indicate that dPAG neurons were more susceptible to being affected by innate fear.

**Fig. 7:**
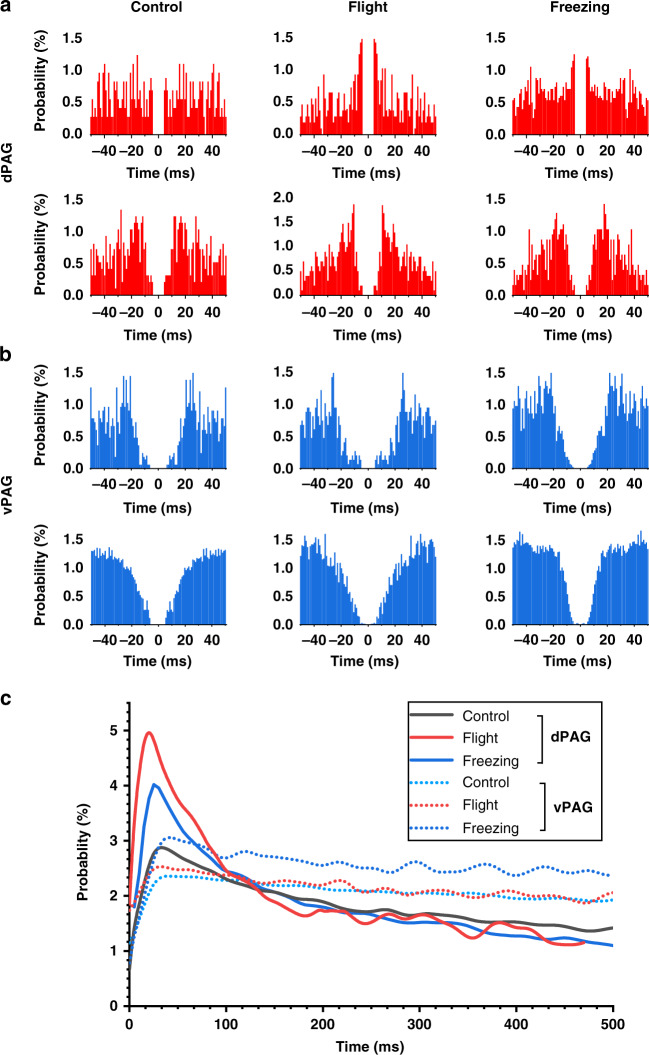
**Statistical analysis of firing characteristics in different states. a** Spike-time autocorrelograms of sample dPAG neurons in different states. **b** Spike-time autocorrelograms of sample dPAG neurons in different states. **c** Mean autocorrelograms of dPAG (*n* = 44) and vPAG (*n* = 40) neurons

The autocorrelograms of PAG neurons displayed behavior-associated firing changes under different defensive responses. dPAG neurons during flight showed a higher probability of firing with short interspike intervals than during control and freezing (Fig. 7a). The distribution of vPAG neuron interspike intervals was relatively more uniform in freezing conditions than in flight and control conditions (Fig. 7b). By pooling all dPAG and vPAG neurons, we also calculated the average autocorrelogram of neurons before and after exposure to 2MT. As shown in Fig. 7c, compared with vPAG neurons, dPAG neurons exhibited a higher probability of firing with short spike-inter intervals. We linearly fit the spike firing rate of dPAG and vPAG neurons to the amplitude and latency of their autocorrelogram peak, respectively. We found that the spike firing rate was positively correlated with the amplitude of the peak value of the autocorrelogram (Fig. S8a) and negatively correlated with the latency of the peak value of the autocorrelogram (Fig. S8b).

These results indicated that a higher amplitude of the autocorrelogram peak and a shorter peak latency predicted a higher neuron firing rate. dPAG and vPAG neurons exhibited changes in firing characteristics under different defense states. The dPAG was more prone to burst or burst-like firing in flight. The spike firing time of vPAG neurons was more evenly distributed in the freezing state so that the vPAG can provide stable and continuous neuroregulatory information to match the long-term freezing behavior.

## Discussion

In this study, we designed an implantable microelectrode array with high spatial and temporal resolution that can be used to record neural information in the dPAG and vPAG of free-moving rats and exposed rats to the highly effective predator analog 2MT to induce innate fear, resulting in flight and freezing. We then recorded and analyzed the neuroelectrophysiological signals and behavioral information in the dPAG and vPAG under defensive behavior. A general consensus has emerged that the dPAG plays a major regulatory role in innate fear^37,38^, whereas the vPAG mainly participates in the integration of conditioned fear^39,40^. The fabricated MEAs were conducive to accurately detecting and synchronously analyzing the neural activities and defensive information transmission of the dPAG and vPAG, avoiding their separation as previously studied. Using MEA as a tool, we found that the dPAG and vPAG were involved in controlling predator odor-induced innate fear and defensive behaviors through electrophysiological data, including flight and freezing. However, dPAG neurons were more robustly activated than vPAG neurons in active defense and transmitting neuronal defense information to the vPAG. The dPAG is responsible for organizing defense behavior against predators and transmitting neural defense information. vPAG neurons were more strongly activated and dPAG activation was decreased when rats stopped movement and were freezing. The dominance of the freezing phase gradually shifted to the vPAG. In this process, defense information was not transmitted between two nuclear clusters, which seemed to be mediated by different pathways. However, after exposure to 2MT, the delta frequency band of the dPAG was more active. In addition, the vPAG was active in the theta frequency band, indicating that the frequency bands of neural regulation between the two were different.

Flight is a short-term and explosive behavior, while freezing is a long-term and stable state. dPAG and vPAG performance drove these two opposite types of behavior as follows: the increase in vPAG activities was slow and matched with the long-term freezing response; the significant increase in dPAG activity was short-lasting, matching the short-term response. Therefore, we further studied the characteristics of dPAG and vPAG neurons to explore their cellular mechanisms. Our research results showed that dPAG neuron spikes had a shorter peak duration and better waveform symmetry, and autocorrelation analysis showed a shorter latency. A shorter peak duration indicated that the swifter alternation of neurons might be related to the rapidly increasing firing pattern of dPAG neurons during flight. The spike peak of vPAG neurons lasts longer, and the refractory period of autocorrelation analysis is longer, which shows that the spike peak can be maintained for a longer time. In addition, changes in the spike average waveforms of dPAG and vPAG neurons were also observed during exposure to the odor of predators. The amplitude of the average waveform peak of dPAG neurons increased more significantly than that of vPAG neurons. By pooling all the detected neurons, we found that the spike firing rate was positively correlated with the peak value of the refractory period in autocorrelation analysis and negatively correlated with the peak latency. On the other hand, the latency of the peak value of autocorrelogram analysis of dPAG neurons became shorter when the rats were exposed to predator odor, while the latency and peak refractory of autocorrelation analysis of vPAG neurons did not change significantly. These results showed that the dPAG and vPAG exhibit different firing characteristics, and dPAG neurons exhibit burst or burst-like firing characteristics that match the short-term flight response.

Previous studies in different species have demonstrated the central role of the amygdala in dealing with predators’ threats^41,42^. Lesions of the medial CEA block fear responses to predator odor^43^. After the amygdala receives the information of predator threat through the olfactory bulb, these signals are conveyed to the dPAG for the organization of defense responses via the medial hypothalamus^44–46^. The process requires the dPAG to respond quickly. The burst firing mode of the dPAG contributes to projecting instructions to the brain stem and other downstream circuits in a short time. In contrast, the CEA directly projects to the vPAG to suppress ongoing motivated behaviors and promote freezing. Consistent with this result, we found that the spike firing rate of vPAG neurons was positively correlated and the spike firing rate of dPAG neurons was negatively correlated with freezing behavior (Fig. S7). These findings revealed that different aspects of defensive responses seemed to be mediated by different pathways involving PAG, which also explained the different activation states of dPAG and vPAG under the same behavior.

## Conclusions

In the present study, we designed and fabricated an implantable neural MEA, which was used to simultaneously detect dynamic neurophysiological activities of PAG double-subregions in rats exhibiting a defensive state. Our results suggested that both dPAG and vPAG neurons were involved in regulating the innate fear response to the predators’ threat. The activation of dPAG neurons reached the highest level in flight, and the activation of vPAG neurons reached the highest level in freezing. Activated dPAG transmitted neural defense information to the vPAG. The application of our MEAs revealed that the different cellular mechanisms of the dPAG and vPAG matched their main regulated behaviors. Furthermore, these results demonstrated that PAG seems to participate in distinct pathways to control different types of defense responses. Our research lays the foundation for further research on the role of PAG in the regulation of congenital fear and precisely targeted treatment of fear-related diseases in the future.

## Supplementary information

Supplementary Information
