# Supplementary material for: Detection of neuronal defensive discharge information transmission and characteristics in periaqueductal gray double-subregions using PtNP/PEDOT:PSS modified microelectrode arrays
Source: Microsyst Nanoeng. 2023 May 31;9:70. doi: 10.1038/s41378-023-00546-8 (PMC10232427; doi:10.1038/s41378-023-00546-8)
Supplement: Supplementary file 1 — Supplementary Information [file 41378_2023_546_MOESM1_ESM.docx]

**Supplementary Information**

Detection of Neuronal Defensive Discharges Information Transmission and Characteristics in the Periaqueductal Gray Double-Subregions Using PtNPs/PEDOT:PSS-modified Microelectrode Arrays

Botao Lu ^[[1]](#footnote-0),^^[[2]](#footnote-1)^, Penghui Fan ^1,2^, Ming Li ^1,2^, Yiding Wang ^1,2^, Wei Liang ^1^, Gucheng Yang ^1,2^, Fan Mo ^1,2^,

Zhaojie Xu ^1,2^, Jin Shan ^1,2^, Yilin Song ^1,2^, Juntao Liu ^1,2,*^, Yirong Wu^1,2,*^ and Xinxia Cai ^1,2,*^

**This file includes:**

Figures S1–S8.


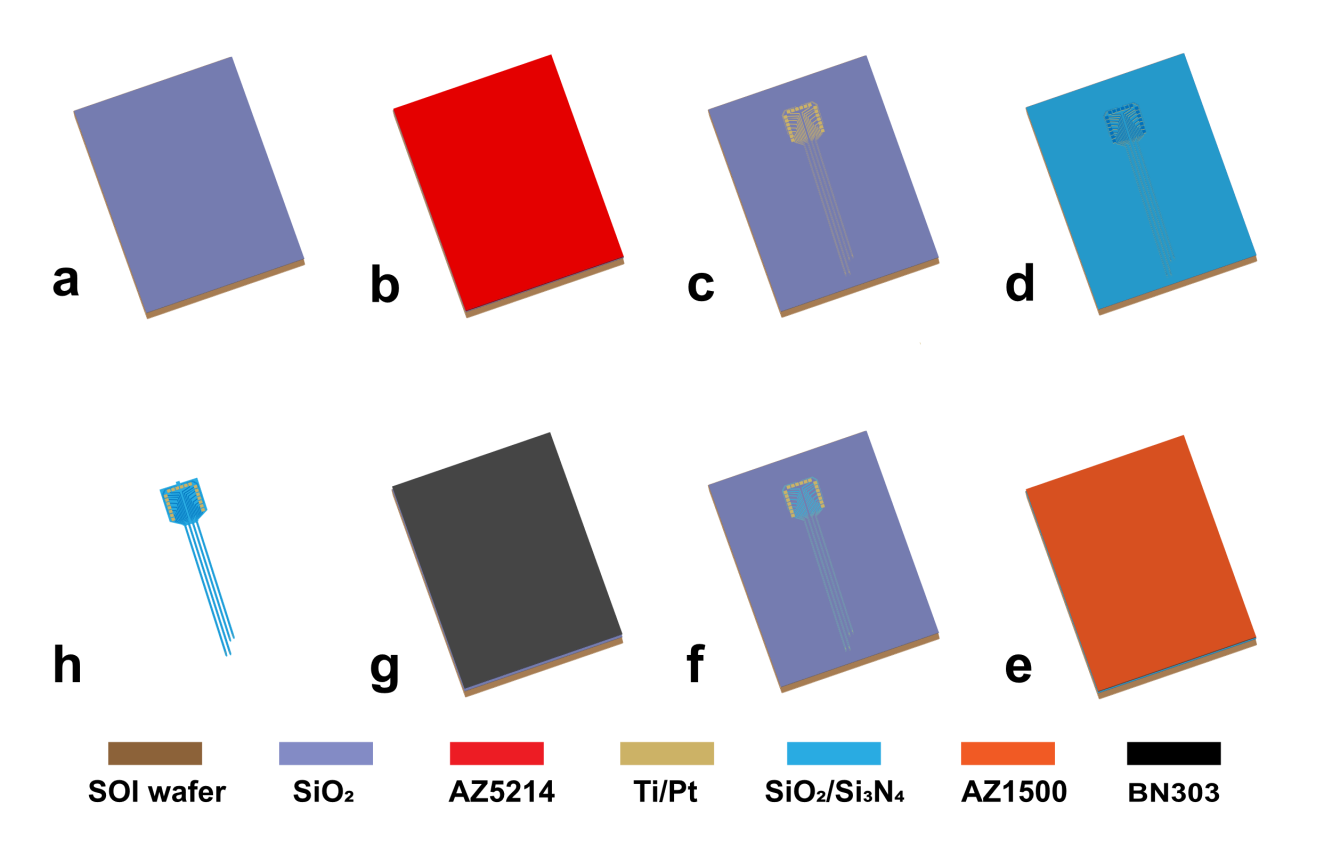


**Figure S1 Fabrication of the MEA. a** A 200-nm layer of SiO_2_ was produced for insulating devices from the SOI substrate. **b** AZ 5214E photoresist was spin coated on wafer. **c** 250 nm Pt and 30 nm Ti were deposited by sputtering as metal layer to Sputter and lift-off to form the metal Pt/Ti layer. **d** 300 nm SiO_2_and 500 nm Si_3_ N_4_ was deposited as insulating layer. **e** AZ 1500 photoresist was spin coated on wafer. **f** Exposure of recording sites and bonding pads. **g** BN303 was spin-coated on wafer. **h** Wet etching to release of the MEA from the substrate.


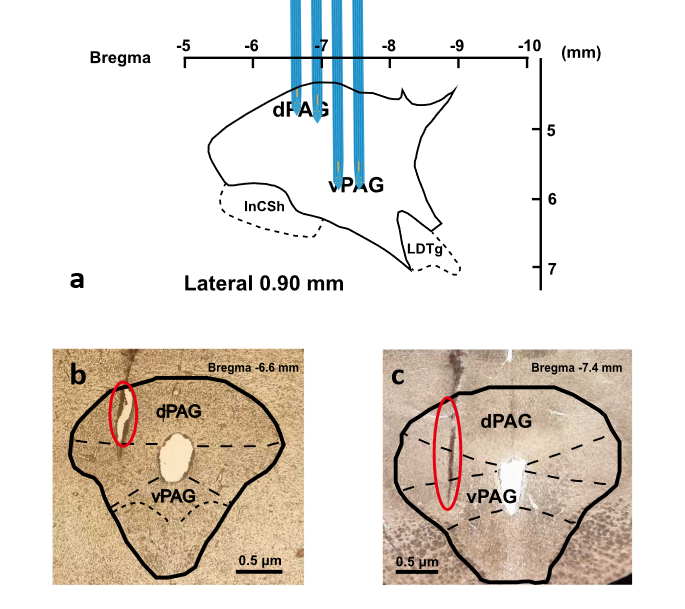


**Figure S2 Implantation position of MEA.** **a** Schematic diagram of MEA implantation position in the midbrain periaqueductal gray (PAG). **b c** The coronal sections of PAG showing the position of different handles of MEA in the same rat PAG. Approximate position of section relative to bregma was indicated. The red mark was the MEA implantation position.


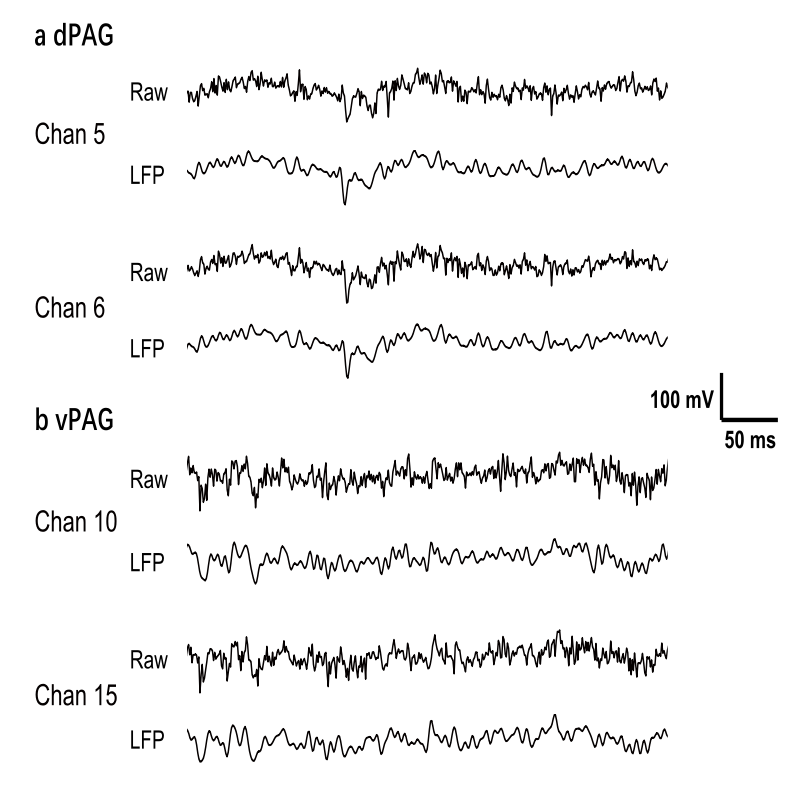


**Figure S3 Comparison of raw data and the local field potential (LFP).** **a** The typical raw data and LFP of dPAG neurons. **b** The typical raw data and LFP of vPAG neurons. The sampling rate of the raw signal was 1 kHz, and the LFP was extracted from the raw data by using a 200 Hz low-pass filter.


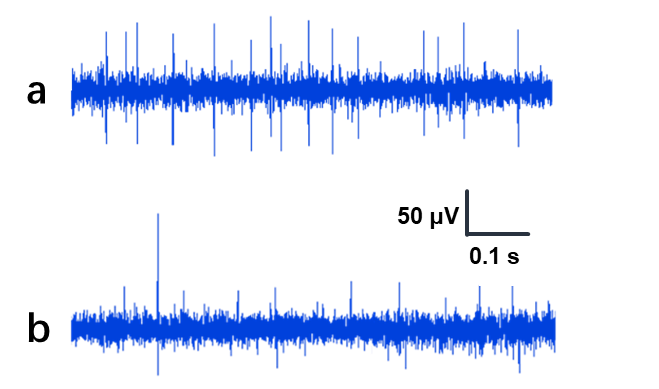


**Figure S4** Typical sample signal after 200Hz high pass filtering recorded by microelectrode modified with PtNPs /PEDOT: PSS at **a** 5 days and **b** 40 days of in vivo recording.


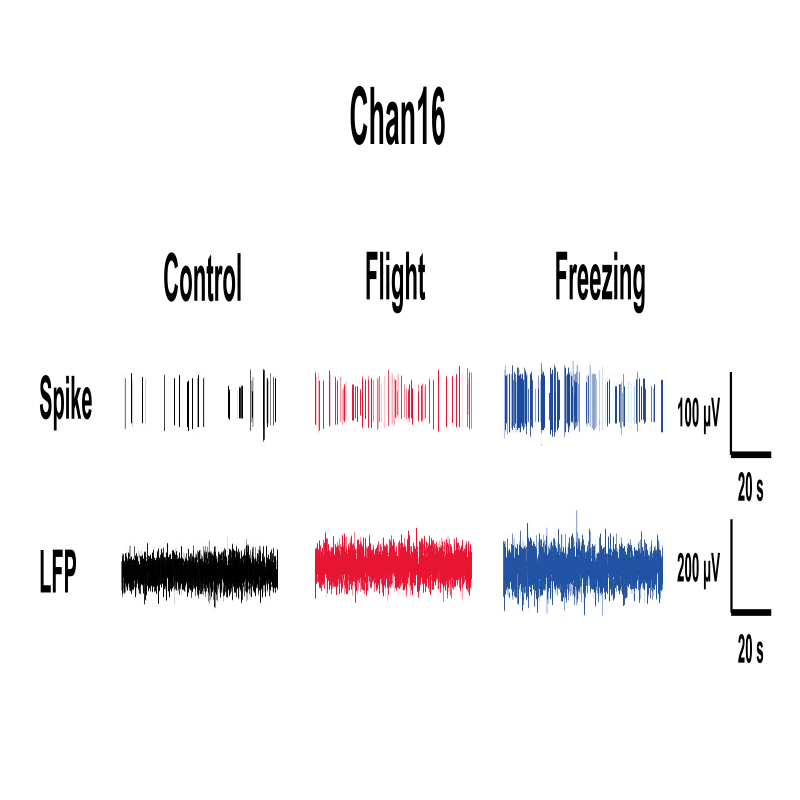


**Figure S5** The real-time recordings of spike and LFP of vPAG neurons (Chan16) during 2MT-induced defense behavior in rats.


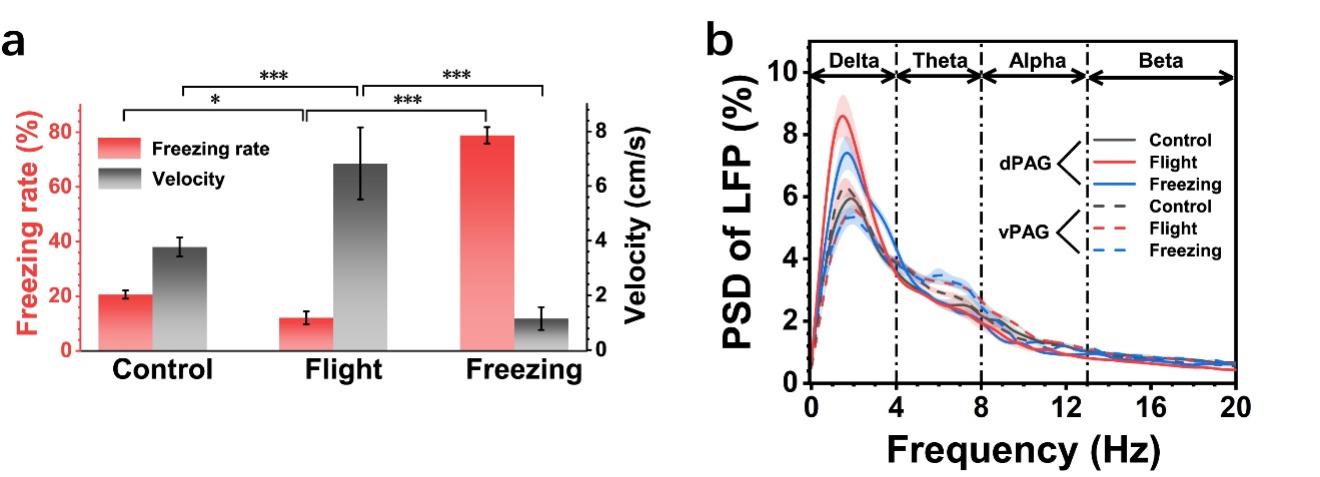


**Figure S6** Quantitative analysis of main parameters of behavior and electrophysiology. **a** Average freezing rate and velocity of rats in different states. **b** Power spectral density change of LFP in 0-20Hz frequency band. The solid line represents dPAG and the dotted line represents vPAG.


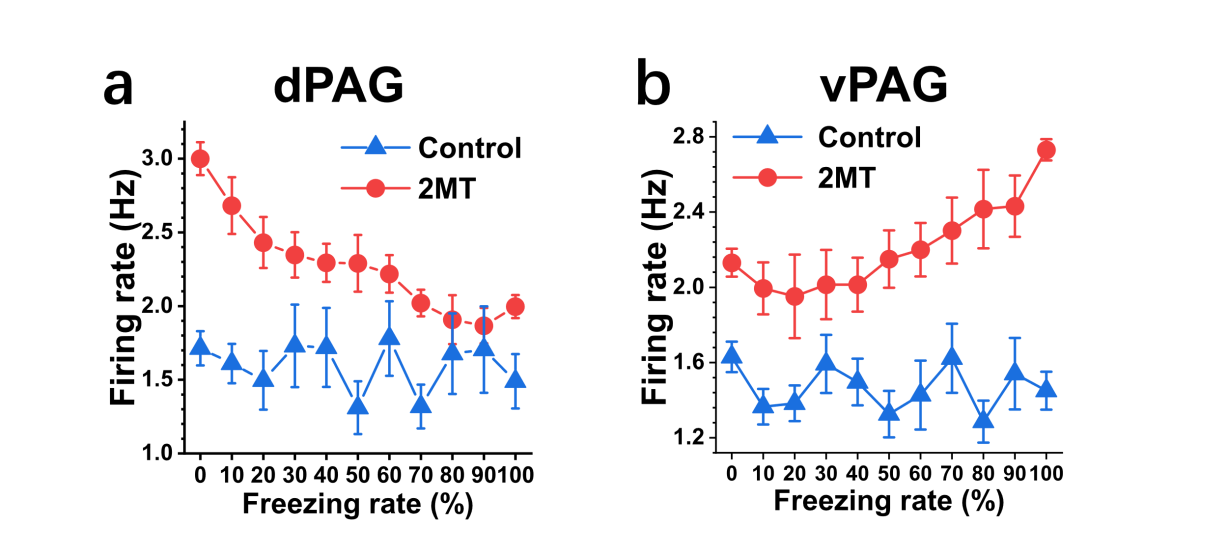


**Figure S7** **The correlation between spike firing rate and freezing rate.** **a** The relationship between dPAG neurons and freezing rate under control and 2MT. **b** The relationship between vPAG neurons and freezing rate under control and 2MT.


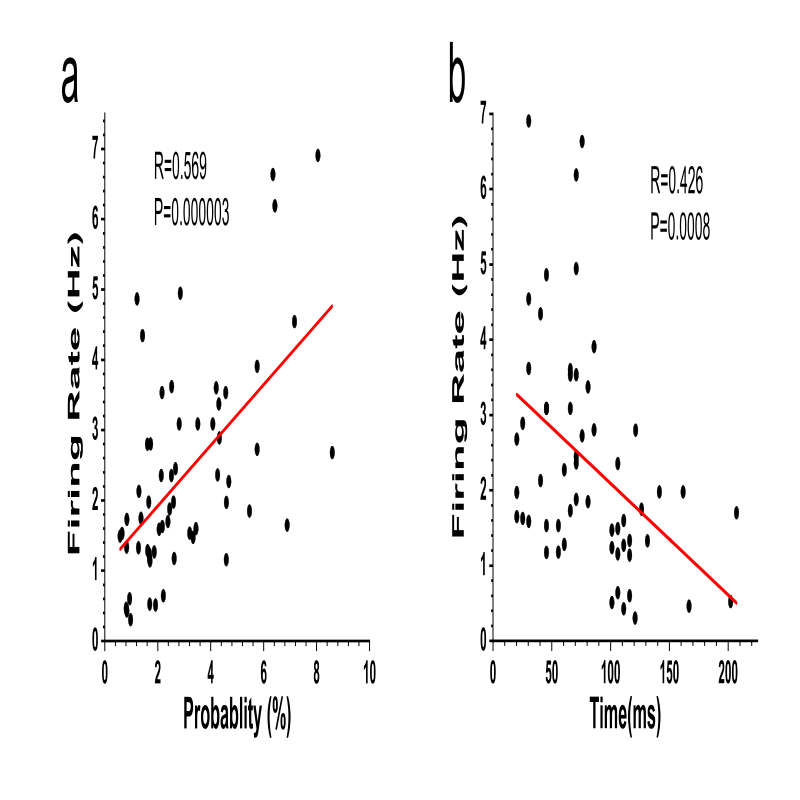


**Figure S8 a** The firing rate of neurons was positively correlated with the amplitude of the autocorrelogram peak exposure to predator odor. **b** The firing change of neurons was negatively correlated with the latency of the autocorrelogram peak.

1. State Key Laboratory of Transducer Technology, Aerospace Information Research Institute, Chinese Academy of Sciences, Beijing 100190, China [↑](#footnote-ref-0)
2. University of Chinese Academy of Sciences, Beijing 100049, China

   * Correspondence: Juntao Liu (liujuntao@mail.ie.ac.cn) or Yirong Wu(wyr@mail.ie.ac.cn) or Xinxia Cai(xxcai@mail.ie.ac.cn) [↑](#footnote-ref-1)
